# Radiographic Evaluation of Implant Stability and Osseointegration in Adult Orthodontic Patients: Radiographic Evaluation of Implant Stability and Osseointegration

**DOI:** 10.31661/gmj.v14i.3799

**Published:** 2025-04-18

**Authors:** Sajjad Rostamzadeh, Mohammad Ghasemirad, Mohammad Gerayeli, Mina Abasi, Mohsen Pouresmaeliyan Roumani, Shabnam Ganjehzadeh, Amirmohammad Moharrami

**Affiliations:** ^1^ Department of Orthodontics, Faculty of Dentistry, Tabriz University of Medical Sciences, Tabriz, Iran; ^2^ Department of Periodontics, Faculty of Dentistry, Rafsanjan University of Medical Sciences, Rafsanjan, Iran; ^3^ Dental Research Center, Mashhad University of Medical Sciences, Mashhad, Iran; ^4^ School of Dentistry, Tehran University of Medical Sciences, Tehran, Iran; ^5^ Private Dental Clinic, Shahrebabak, Kerman,Iran; ^6^ Department Of Orthodontics, Faculty of Dentistry, Tehran University of Medical Sciences, Tehran, Iran

**Keywords:** Implant Stability, Osseointegration, CBCT, Orthodontic Mini-implants, Temporary Anchorage Devices (TADs), Artificial Intelligence, Peri-implant Bone Loss

## Abstract

Radiographic evaluation is essential for assessing implant stability and osseointegration in adult orthodontic patients. The success of temporary anchorage devices (TADs) and mini-implants depends on primary stability, achieved through mechanical engagement, and secondary stability, influenced by bone remodeling. While traditional clinical methods, such as mobility testing, provide subjective assessments, radiographic imaging offers objective insights into bone-implant interactions. Periapical and panoramic radiographs are commonly used but are limited by their two-dimensional (2D) nature. Cone beam computed tomography (CBCT) has emerged as the gold standard, providing three-dimensional (3D) visualization of cortical bone thickness, marginal bone loss, and peri-implant adaptations. However, challenges such as image artifacts, radiation exposure, and observer variability persist. Implant stability is influenced by factors like bone density, cortical thickness, insertion torque, and patient-specific variables, including systemic conditions, genetic predisposition, and lifestyle habits. Emerging techniques such as resonance frequency analysis (RFA) complement radiographic findings by providing quantitative stability assessments. Additionally, artificial intelligence (AI)-driven radiographic analysis is improving diagnostic accuracy, automating bone density evaluation, and predicting implant success. Future advancements in low-dose CBCT protocols, AI-assisted diagnostics, and digital treatment planning aim to optimize implant placement and long-term stability assessment. By integrating multimodal imaging approaches with biomechanical and AI-driven predictive modeling, clinicians can enhance treatment planning, reduce implant failure rates, and improve orthodontic outcomes. This review underscores the importance of advanced imaging techniques in implant stability assessment and highlights the need for continued research in AI-driven diagnostics and minimally invasive evaluation methods.

## Introduction

Implant stability and osseointegration are fundamental determinants of the success of dental implants in orthodontic applications [1]. The increasing use of temporary anchorage devices (TADs) and orthodontic mini-implants has revolutionized treatment modalities, providing predictable anchorage with minimal patient compliance [2].

However, the long-term success of these implants is contingent upon their ability to achieve and maintain stability within the bone [3]. A key factor influencing implant longevity is the process of osseointegration, which is characterized by the direct structural and functional connection between bone and implant surface [4]. Given the dynamic biomechanical forces present in orthodontic treatment, the assessment of implant stability is particularly critical [5].

Radiographic evaluation plays a pivotal role in assessing implant stability and osseointegration, offering objective insights into bone-implant interactions [6]. Traditional clinical methods, such as tactile assessment and mobility testing, provide only subjective and qualitative data, often failing to detect early complications or subtle peri-implant changes [7].

Advanced imaging techniques, including periapical and panoramic radiographs, as well as cone beam computed tomography (CBCT), have significantly improved the ability to assess marginal bone levels, cortical engagement, and bone density changes over time [1]. While periapical radiographs offer high-resolution imaging for localized bone evaluation, they are limited by their two-dimensional (2D) nature, which may obscure buccal and lingual bone changes [5].

Similarly, panoramic radiographs, though useful for broad anatomical assessment, have been criticized for their inherent distortion and lower accuracy in detecting early peri-implant bone loss [8].

Recent studies emphasize the role of CBCT in providing three-dimensional (3D) visualization, allowing for a more precise assessment of implant positioning and peri-implant bone adaptations [2]. For instance, Cui et al. [9] provided valuable insights into the variations in crestal soft tissue thickness using this technique ; their findings revealed only a weak correlation between soft and hard tissue measurement. However, the study's retrospective design and limited anterior site sample size may restrict its clinical applicability [9]. Additionally, image artifacts from metallic restorations may affect CBCT accuracy, requiring further refinement of metal artifact reduction algorithms to ensure reliable diagnostics [7][10]. While this technique remains the most comprehensive imaging modality for implant evaluation, its use should be strategically balanced against radiation exposure risks, particularly in routine monitoring [5].

The importance of radiographic evaluation extends beyond initial implant placement, playing a crucial role in predicting implant success [11].

Bone remodeling, a continuous process of resorption and deposition, can significantly influence implant stability over time [4] Factors such as implant loading conditions, bone quality, and patient-specific variables contribute to variations in osseointegration, necessitating longitudinal radiographic monitoring [5].

Early detection of bone loss or peri-implant radiolucency allows for timely intervention, preventing implant failure [1]. Furthermore, the integration of artificial intelligence (AI) in radiographic assessment has opened new avenues for automated analysis, improving diagnostic precision and reducing observer variability [6].

Several radiographic modalities are currently employed to evaluate implant stability, each offering distinct advantages and limitations. Periapical radiographs, widely used for assessing marginal bone levels, provide high-resolution imaging but are limited by their two-dimensional (2D) nature[3][5]. Panoramic radiographs offer a broader field of view, facilitating overall treatment planning but with reduced image sharpness [2]. CBCT, as the gold standard for 3D imaging, enables comprehensive assessment of bone volume, cortical engagement, and implant angulation[11]. This review aims to provide a comprehensive analysis of radiographic techniques used for evaluating implant stability and osseointegration in adult orthodontic patients.

## Biomechanics of Implant Stability in Orthodontics

Implant stability is a fundamental requirement for the success of orthodontic implants, including TADs and mini-implants. Stability ensures the implant’s ability to resist micromovements under functional loads, which is critical for achieving predictable orthodontic [12]. Implant stability is classified into two distinct phases: primary stability, which is mechanically achieved at the time of insertion, and secondary stability, which results from biological remodeling and osseointegration over [13]. A clear understanding of the biomechanical principles governing implant stability is essential for optimizing clinical protocols, improving implant success rates, and minimizing failure risks in orthodontic patients [12].

## Primary vs. Secondary Stability

Primary stability is the initial mechanical engagement of the implant with the surrounding bone, primarily dependent on bone quality, implant geometry, and insertion technique [13]. In orthodontic applications, mini-implants and TADs rely heavily on cortical bone engagement to achieve sufficient primary stability [14][15]. Studies have shown that high insertion torque (>10 Ncm) is associated with improved primary stability, as it ensures adequate bone-implant contact and reduces micromovements that could disrupt osseointegration[16][17].

However, excessive torque may cause microdamage to the bone, potentially leading to implant failure [18].

Secondary stability develops over time as a result of biological processes, including bone remodeling and osseointegration [19]. Unlike endosseous dental implants, which rely heavily on osseointegration for long-term stability, orthodontic mini-implants often function through mechanical retention without complete osseointegration. Nevertheless, peri-implant bone adaptation and remodeling influence implant longevity. A study by Monje et al.[12] demonstrated that increased cortical bone thickness enhances secondary stability by providing greater resistance to micromovements, thereby reducing the risk of early implant failure. This highlights the importance of patient-specific bone characteristics in treatment planning [12].

The transition from primary to secondary stability is a critical period where implant micromovements must be minimized to prevent fibrous encapsulation, which can lead to implant loosening [20]. If excessive mobility exceeds 50-150 µm, the formation of a fibrous interface instead of direct bone contact may occur, compromising implant retention [21]. This underscores the significance of controlled loading conditions during the early phases of implantation [22].

## Factors Influencing Implant Stability

**Table:** Table1. Common Factors Affecting Implant Stability

**Factor**	**Influence on Stability**	**Clinical Consideration**
Bone Quality	Higher density bone increases primary stability.	Preoperative assessment (CBCT), potential bone grafting for low-density bone.
Bone Quantity	Sufficient bone volume ensures better implant anchorage.	Ridge augmentation or sinus lift may be required in atrophic ridges.
Implant Design	Thread design and surface roughness enhance mechanical stability.	Selection based on patient-specific bone conditions.
Implant Length	Longer implants provide greater surface area for osseointegration.	Avoid excessive length near anatomical structures (e.g., nerves, sinuses).
Implant Diameter	Wider implants distribute occlusal forces better.	Limited by available bone width; risk of cortical bone resorption.
Surgical Technique	Precise osteotomy and insertion torque affect primary stability.	Minimize trauma, consider guided surgery for precision.
Immediate vs. Delayed Loading	Immediate loading can reduce stability if not well-planned.	Assess primary stability; delay loading if needed.
Occlusal Forces	Excessive loading can cause micro-movements and implant failure.	Proper occlusal adjustments, use of splints if necessary.
Host Factors (Systemic Conditions)	Conditions like osteoporosis or diabetes may impair osseointegration.	Preoperative screening and medical management before surgery.
Smoking & Medications	Smoking and certain drugs (e.g., bisphosphonates) reduce bone healing.	Encourage smoking cessation; review patient medication history.

Table-1 demonstrated common factors affecting implant stability. Implant stability is fundamentally influenced by bone quality, design parameters, and biomechanical loading [23]. Denser bone with greater cortical thickness provides superior mechanical interlocking, making it a critical factor for both conventional implants and TADs [24]. Regions such as the adult mandible typically offer enhanced primary stability due to their dense and thick cortical structure. In contrast, sites with low-density trabecular bone or thin corticeslike the posterior maxilla or in adolescent patients often yield lower insertion torque and higher failure rates[23][25][26].

Lee et al [25]. found that cancellous bone density had a greater effect on miniscrew success than cortical thickness alone. Also, Truong et al [27]. similarly emphasized bone mineral density as the key determinant in TAD migration under load. Additionally, implant design contributes substantially for TADs, longer screws (≥8 mm) and diameters of 1.5-1.6 mm optimize retention while minimizing cortical trauma [27]. Tapered screw geometries yield higher primary stability than cylindrical designs [28], while thread design and insertion technique (self-drilling vs. pre-drilling) are less influential [27].

In conventional implants, tapered and hybrid geometries outperform parallel-walled implants in soft bone [29][30]. However, surface roughness primarily supports osseointegration, not immediate mechanical stability [29]. Loading protocols and biomechanical factors further influence outcomes. Immediate loading, placing the prosthetic shortly after implant placement, has shown comparable survival rates to delayed loading when primary stability is sufficient (insertion torque ≥30-35 Ncm or ISQ ≥60) [31]. Orthodontic mini-screws, in contrast, are usually loaded immediately and require careful attention to force magnitude and duration [32]. Excessive insertion torque or omitting pilot drilling can cause cortical microdamage, reducing initial retention [33]. Truong et al. reported that bone trauma during insertion compromises early stability , though pilot drilling with smaller diameters can mitigate this risk [27]. Bicortical anchorage techniques improve resistance to displacement over time compared to monocortical anchorage [34], but even well-placed screws exhibit some degree of "creep" under sustained load [35]. Long-term success also depends on biological integration. Conventional implants experience an initial dip in stability as bone remodels, then recover through secondary stability supported by surface roughness [36].

Finally, patient-specific factors such as age, systemic health, oral hygiene, and anatomical site must be considered. Stability is generally lower in younger patients with immature bone [37], and conditions like diabetes and osteoporosis impair osseointegration [38]. Inflammatory conditions, poor hygiene, and unfavorable soft tissue can also compromise stability[24]. Customized planning using 3D imaging is therefore essential to identify optimal insertion sites, avoid anatomical risks, and maximize primary retention [39].

## Radiographic Techniques for Evaluating Implant Stability

**Table:** Table2. Radiographic Techniques for Implant Stability

**Imaging Modality**	**Primary Use**	**Strengths**	**Limitations**	**Clinical Applicability**
**Periapical Radiography**	Monitoring crestal bone and implant threads	High spatial resolution; low cost; low radiation	2D only; distortion and superimposition; no buccolingual detail	Routine follow-up; baseline and longitudinal records
**Panoramic Radiography**	Full arch overview	Broad coverage; quick; useful for planning	Distortion; low resolution; limited detail near implant sites	Initial evaluation; surgical planning context
**CBCT**	3D assessment of peri-implant bone	Volumetric analysis; accurate bone thickness and density estimation	Metal artifacts; variable dose; cost; limited HU calibration	Pre-surgical planning and postoperative evaluation
**Micro-CT**	Research-level assessment of osseointegration	Ultra-high resolution; 3D evaluation of bone-implant interface	High radiation; ex vivo only; expensive	Preclinical studies; experimental models
**Dynamic Digital Radiography (DDR)**	Functional assessment (experimental)	Potential to monitor micro-motion and bone remodeling under load	Still investigational; not yet validated for dental implants	Future application in real-time implant stability testing
**Spectral/Photon-Counting CT**	Artifact reduction; bone quality mapping	Reduces metal artifacts; high-resolution and contrast	High cost; limited clinical availability in dentistry	Emerging; promising for enhanced peri-implant evaluation

Accurate assessment of implant stability is crucial for ensuring long-term success in implant dentistry [40]. Radiographic techniques provide essential information on peri-implant bone levels, implant positioning, and potential complications. Various imaging modalities differ in their resolution, depth of information, and clinical application [41]. Selecting the appropriate technique depends on the stage of treatment, the complexity of the case, and the required diagnostic precision [42]. Table-2 shows the comparation of current and future radiographic techniques for Implant Stability.

### Periapical Radiography

Periapical Radiography is widely used for early implant evaluation due to its high resolution and ability to detect marginal bone loss. It provides detailed imaging of the implant and surrounding bone, making it suitable for monitoring crestal bone changes [43]. However, its two-dimensional (2D) nature limits its ability to assess buccal and lingual bone defects. Despite these limitations, periapical radiographs remain a primary tool in routine follow-ups, particularly in cases where vertical bone loss is of concern [44].

### Panoramic Radiography

Panoramic Radiography offers a broader field of view, making it useful for evaluating multiple implants, adjacent anatomical structures, and overall bone levels [45]. It provides essential preoperative information for implant planning but lacks the fine detail required to assess early peri-implant changes accurately. The inherent distortion and magnification errors associated with panoramic images can affect precision; thus, they are often complemented by other imaging methods for more detailed assessments [44].

### CBCT

CBCT is now widely used in implant dentistry to overcome many limitations of planar radiographs. This technique generates true 3D volumetric images of the jaws, allowing visualization of the bone implant interface in all dimensions [46]. Unlike 2D films, CBCT can measure bone thickness and volume around implants, detect buccal or lingual dehiscences, and identify peri-implant defects that lie outside the narrow field of a periapical film [47]. Its superiority over conventional 2D radiographs lies in its ability to detect buccal and lingual cortical bone changes, which are critical in assessing implant stability [48]. However, the higher radiation dose compared to traditional radiographs necessitates judicious use, particularly in follow-up assessments [46].

## Clinical Application and Challenges of Radiographic Techniques

**Figure-1:**
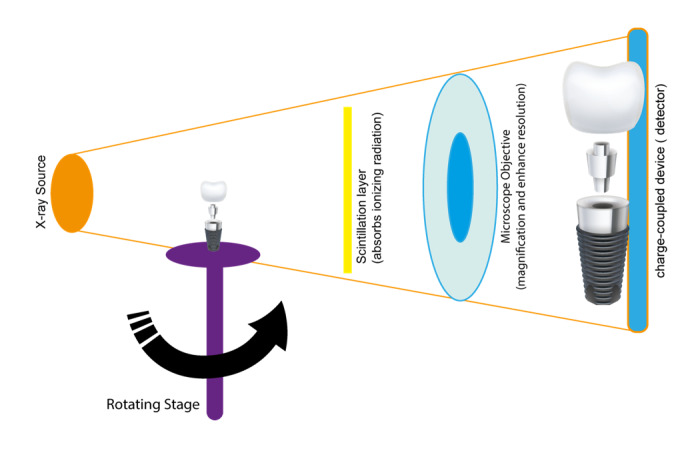
Figure 1. schematic illustration of micro-CT technique

**Figure-2:**
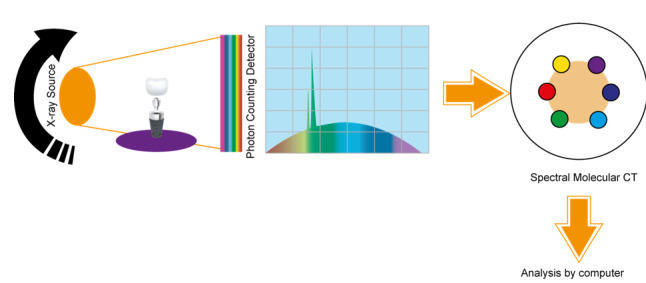
Figure 2. photon-counting CT Imaging proceeding

Radiographic imaging is a cornerstone of clinical management for both temporary orthodontic implants (TADs/mini-screws) and conventional dental implants. Clinicians routinely use intraoral and panoramic radiographs to verify implant position and to monitor marginal bone levels over time, while CBCT) provides detailed three-dimensional assessment of bone anatomy and implant orientation [43][49]. In orthodontic cases, CBCT also aids in planning mini-implant placement by identifying interradicular bone volume and root positions; studies show that CBCT-guided placement yields higher accuracy and fewer root perforations than two-dimensional radiographs alone [50][51]. After insertion, radiographic follow-up is used to detect signs of osseointegration or instability. Conventional implants are monitored for crestal bone loss (an indicator of peri-implant health) and any radiolucency at the implant interface [51].

Despite these applications, radiographic evaluation faces several important challenges:

• Technical imaging limitations: Two-dimensional images suffer from overlap and distortion. Panoramic and periapical films cannot depict the bucco-lingual dimension and may compress interradicular spaces, making depth assessment difficult [50]. While CBCT overcomes many projection issues by providing multi-planar 3D views, it introduces other limitations [52]. CBCT resolution, although high, still does not resolve the microscopic bone implant interface even sophisticated micro-CT and synchrotron imaging studies indicate that the first few millimeters of bone contact cannot be distinguished on clinical radiographs [53] . Metal artifacts are a further problem; orthodontic brackets, wires or implant fixtures can create streaks and noise that obscure peri-implant bone details [49]. Moreover, there is no universal standard for image acquisition and calibration. Radiographic gray values (in CBCT or digital X-rays) are not directly comparable across machines or settings, complicating attempts to quantify bone density or detect subtle changes [49]. Low-dose CBCT protocols have been developed to reduce radiation, but lowering dose often comes at the expense of image quality and contrast, potentially impairing the detection of early bone changes [49][54].

• Diagnostic interpretation variability: Reading radiographs is subjective and prone to observer error. Studies of peri-implant bone measurements report inter- and intra-observer differences on the order of 0.3-0.5 mm or more [55]. Such variability approaches the minimal clinically important change (≈0.2 mm/year of bone loss) and thus can mask early pathological bone loss or falsely suggest stability [55].

• Patient-related and workflow barriers: Practical constraints also limit radiographic evaluation. CBCT imparts a higher radiation dose than conventional films, so its use must be judicious. In orthodontic patients providers reserving CBCT for cases where the added diagnostic benefit justifies the dose [49][56] Even when CBCT is indicated (e.g. implant in a densely crowded area), obtaining a scan requires additional chair time and expense, and not all orthodontic offices have easy access to in-house CBCT [57].

• Orthodontic appliances themselves can complicate imaging: brackets and wires may need to be removed or repositioned to avoid artifact on a CBCT scan. Patient movement is another issue; children or anxious patients may be less compliant during the longer acquisition time of a 3D scan [58]. Workflow-wise, routine implant planning (especially for mini-implants) still often relies on quick 2D radiographs for efficiency [59]. Finally, since TADs are temporary, clinicians may not schedule multiple follow-up radiographs specifically to assess osseointegration, unlike permanent implants, which can delay detection of complications until clinical symptoms appear [60].

## Emerging Technologies and Future Directions

### AI and Machine Learning

Deep learning algorithms can now automatically analyze dental radiographs and CBCT scans to detect subtle changes around implants. The performance of the AI models demonstrated considerable variability [61]. Reported overall accuracy ranged from 61.0% to 94.74%, while precision values varied substantially, from as low as 0.63% to as high as 100%. Sensitivity values ranged between 67.0% and 94.44%, whereas specificity ranged from 87.0% to 100% [62]. Integration of AI into clinical workflows also requires standardized datasets and robust validation to ensure consistent performance in routine orthodontic practice [61].

### Micro-computed Tomography (micro-CT)

Micro-CT has emerged as a pivotal imaging modality in dental implantology, offering unparalleled insights into bone microarchitecture and implant integration [63] Figure-1 shows the schematic illustration of micro-CT technique [64].

Its high-resolution, three-dimensional imaging capabilities enable detailed assessments of osseointegration, bone volume, and trabecular morphology, which are critical factors for implant success [65]. Recent studies have demonstrated that micro-CT provides superior spatial resolution compared to conventional imaging techniques, such as CBCT and intraoral radiography [63].

This enhanced resolution allows for precise quantification of bone volume fraction (BV/TV), trabecular thickness (Tb.Th), and bone-implant contact (BIC), facilitating a comprehensive evaluation of implant stability and osseointegration [66]. For instance, a systematic review highlighted that micro-CT enables accurate measurements of bone microstructure parameters, which are essential for predicting implant success and longevity [63].

Despite its advantages, the clinical application of micro-CT is limited due to factors such as high radiation exposure, cost, and the necessity for ex vivo analysis [67].

Consequently, its use is predominantly confined to preclinical studies and in vitro assessments [68]. In summary, while micro-CT currently serves as a valuable tool in research settings for evaluating implant stability and osseointegration, further technological advancements are necessary to overcome existing barriers to its routine clinical use [63].

### Spectral and Photon-counting CT Imaging

Energy-resolved imaging is an emerging frontier for implant evaluation. Dual-energy CT or CBCT (DECT/DE-CBCT) and photon-counting CT (PCCT) exploit X-ray spectra to improve material discrimination. In phantom studies, dual-energy CBCT has been shown to generate virtual monoenergetic images that significantly reduce metal-induced artifacts around dental implants [69]. Figure-2 illustrate the photon-counting CT Imaging proceeding.

More recently, clinical photon-counting CT scanners (with energy-resolving detectors) have demonstrated sub-millimeter spatial resolution (down to ~100 µm) and inherently reduced metal artifacts when reconstructing images at high virtual energies [69][70]. For instance, photon-counting CT combined with iterative reconstruction achieved crisper implant detail and higher contrast than conventional CBCT [70] These spectral techniques could, in principle, provide more accurate bone density and composition measurements around orthodontic anchorage implants[71][72]. Ongoing research is evaluating whether specialized dental CBCT devices can incorporate spectral filtration or energy discrimination in a dose-efficient manner.

### Advanced Artifact Reduction and Image Quality Enhancement

Metal artifacts remain a major limitation of radiographic implant evaluation. Recent innovations focus on improved reconstruction and post-processing. Traditional iterative metal-artifact reduction (MAR) algorithms (e.g., projection interpolation) have been supplemented by AI-based denoising [73]. Radiologists in that study also rated the AI-filtered CBCT images as having much lower artifact severity. In practical terms, such noise-reduction could help clinicians better visualize the bone-implant interface despite the presence of brackets or retainers. Additionally, spectral imaging techniques inherently combat artifacts: virtual monoenergetic reconstructions (available in dual-energy or photon-counting CT) reduced beam-hardening around implants [71][72]. Overall, these advances suggest that future CBCT systems may deliver much cleaner images around metal. Yet, clinical deployment will require integration of vendor-specific software and validation that bone measurement accuracy is not altered by the artifact correction.

### Radiomics and Quantitative Image Biomarkers

Radiomics, the high-throughput extraction of quantitative image features, offers a data-driven route to characterize peri-implant bone beyond visual assessment. In dental research, radiomic texture analysis has been used to cluster bone quality.

Troiano et al. [74] demonstrated that unsupervised clustering of CBCT-derived radiomic features from edentulous ridges yielded reproducible stratification of bone types, whereas traditional Lekholm-Zarb qualitative classification showed poor inter-observer agreement [74].

This suggests radiomics could provide an objective metric of bone density or structure relevant to implant stability. Similarly, machine learning models combining CBCT intensity patterns with other clinical data have been proposed to predict insertion torque or resonance frequency of implants. Such quantitative approaches may eventually allow radiographs to serve as "biosensors" of osseointegration [61]. Standardization of acquisition protocols and feature pipelines will be critical before radiomic biomarkers can be clinically adopted for orthodontic implants.

### Functional and Dynamic Radiographic Techniques

Beyond static imaging, novel "functional" radiographic methods are being explored. Dynamic digital radiography (DDR), high-frame-rate X-ray sequences, has recently been used in spine and joint imaging to visualize motion or stability under movement [75]. In theory, a similar approach could monitor micro-motion of orthodontic implants under load or capture real-time bone remodeling during orthodontic force application [75]. Another concept is four-dimensional (4D) CBCT, capturing volumetric sequences (e.g., multiple low-dose CBCT over time) to observe bone changes, but radiation dose currently prohibits routine use [76]. To date, these dynamic methods remain experimental; no clinical studies have yet applied DDR or time-resolved CBCT specifically to orthodontic anchorage implants. Nonetheless, future research may adapt low-dose cine-radiography or combine ultrasound with X-ray sensors to provide real-time feedback on implant stability and bone response [77].

## Conclusion

Radiographic evaluation remains essential in assessing implant stability and osseointegration among adult orthodontic patients, providing indispensable diagnostic insights that guide clinical decision-making. While conventional imaging techniques such as periapical and panoramic radiographs continue to be routinely used due to their accessibility and low radiation exposure, CBCT has significantly advanced clinical capability by providing comprehensive three-dimensional analysis of bone structures around implants. Nevertheless, inherent limitations such as metal artifacts, diagnostic variability, and patient-specific workflow barriers persist, highlighting the need for continual refinement.

Emerging imaging technologies and innovative analytical techniques such as artificial intelligence-driven diagnostics, spectral and photon-counting CT, advanced artifact-reduction algorithms, and radiomics are poised to overcome many current challenges. These promising methods have demonstrated superior sensitivity, artifact reduction, and quantitative capability, potentially allowing earlier detection of peri-implant bone changes and more precise implant stability assessments. Despite the exciting possibilities offered by these advancements, widespread clinical adoption requires further validation through robust prospective trials and technological standardization.

Future research should focus on rigorous clinical testing and cross-platform validation of these novel imaging methodologies to fully realize their potential benefits. Ultimately, integrating these advanced technologies into routine orthodontic and implant dentistry practices will enhance diagnostic accuracy, optimize patient outcomes, and drive significant progress in managing implant stability and osseointegration.

## Conflict of Interest

None.

## References

[R1] Hristov IG (2022). IMPLANT DESIGN FACTORS THAT AFFECT PRIMARY STABILITY AND
OSSEOINTEGRATION. Восточно-Европейский Научный Журнал.

[R2] Suzuki S, Kobayashi H, Ogawa T (2013). Implant stability change and osseointegration speed of
immediately loaded photofunctionalized implants. Implant Dentistry.

[R3] Kumar GK, Priya S, Arunmozhi U, Kadhiresan R, Cynthia JRA (2021). Primary implant stability: A leap towards successful
osseointegration – A narrative review. Journal of Indian Dental Association Madras.

[R4] Goharian A (2019 [cited 2025 Mar 6]). Osseointegration of Orthopaedic Implants [Internet]..

[R5] Kittur N, Oak R, Dekate D, Jadhav S, Dhatrak P (43,). Dental implant stability and its measurements to improve
osseointegration at the bone-implant interface: A review. Vol.

[R6] Tabassum S, Murtaza A, Ali H, Uddin ZM, Zehra SS (2017). Finite element analysis (FEA) of dental implant fixture for
mechanical stability and rapid osseointegration. AIP Conference Proceedings.

[R7] Stocchero M (2018 [cited 2025 Mar 6]). On Influence Of Undersized Implant Site On Implant Stability And
Osseointegration [Internet].

[R8] Anil S, Anand PS, Alghamdi H, Jansen JA (2011). Dental implant surface enhancement and osseointegration. Implant dentistry-a rapidly evolving practice.

[R9] Cui X, Reason T, Pardi V, Wu Q, Martinez Luna (2022). CBCT analysis of crestal soft tissue thickness before implant
placement and its relationship with cortical bone thickness. BMC Oral Health.

[R10] Abdelatef M, Fahmy M, Ahmed G (2024). Efficiency of concentrated growth factors on immediate implant
stability and osseointegration in the posterior mandible (Randomized
Controlled Clinical Trial). Alexandria Dental Journal.

[R11] Kheder D, Hayder A (2019). Evaluation of osseointegration of dental implant with and without
primary stability: An experimental study on sheep. Erbil Dental Journal.

[R12] Monje A, Roccuzzo A, Buser D, Wang HL (2023). Influence of buccal bone wall thickness on the peri-implant hard
and soft tissue dimensional changes. A systematic review.

[R13] Uemura M, Motoyoshi M, Yano S, Sakaguchi M, Igarashi Y, Shimizu N (2012). Orthodontic mini-implant stability and the ratio of pilot hole
implant diameter. The European Journal of Orthodontics.

[R14] Kim J, et al (2005). Primary stability and osseointegration of orthodontic
mini-implants. American Journal of Orthodontics and Dentofacial Orthopedics.

[R15] Miyawaki S, et al (2003). Factors influencing stability of orthodontic mini-implants. American Journal of Orthodontics and Dentofacial Orthopedics.

[R16] Greenstein G, Cavallaro J (2017). Implant Insertion Torque: Its Role in Achieving Primary Stability
of Restorable Dental Implants. Compend Contin Educ Dent.

[R17] Dkheel IA, Al-Quisi A, AlOtaibi NM (2024). The reliability of insertion torque as an indicator for primary
stability in immediate dental implant: A prospective clinical study. J Baghdad Coll Dent.

[R18] Barone A, Alfonsi F, Derchi G, Tonelli P, Toti P, Marchionni S, et al (2016). The Effect of Insertion Torque on the Clinical Outcome of Single
Implants: A Randomized Clinical Trial. Clin Implant Dent Relat Res.

[R19] Soto-Peñaloza D, Martín-de-Llano JJ, Carda-Batalla C, Peñarrocha-Diago M, Peñarrocha-Oltra D (2019). Basic Bone Biology Healing During Osseointegration of Titanium
Dental Implants. Atlas of Immediate Dental Implant Loading.

[R20] Upadhyay MA, Nanda RA (2020). Biomechanics principles in mini-implant driven orthodontics
Temporary Anchorage Devices in Orthodontics (Second Edition). St Louis Elsevier.

[R21] Vladareanu L, Capitanu L (2012). Hybrid force-position systems with vibration control for
improvement of hip implant stability. Journal of Biomechanics.

[R22] Upadhyay M, Yadav S, Nagaraj K, Patil S (2008). Treatment effects of mini-implants for en-masse retraction of
anterior teeth in bialveolar dental protrusion patients: A randomized
controlled trial. American Journal of Orthodontics and Dentofacial Orthopedics.

[R23] De Elío, Del Canto, Del Canto, Orea CJ, Del Canto, Calvo JS (2020). Alveolar Bone Density and Width Affect Primary Implant
Stability. J Oral Implantol.

[R24] Al-Juboori H, Petronis Z, Razukevicius D (2024). The Interrelation between Cortical Bone Thickness and Primary and
Secondary Dental Implant Stability: a Systematic Review. J Oral Maxillofac Res.

[R25] Lee DW, Park JH, Bay RC, Choi SK, Chae JM (2021). Cortical bone thickness and bone density effects on miniscrew
success rates: A systematic review and meta-analysis. Orthod Craniofac Res.

[R26] Fernández-Olavarria A, Gutiérrez-Corrales A, González-Martín M, Torres-Lagares D, Torres-Carranza E, Serrera-Figallo MÁ (2023). Influence of different drilling protocols and bone density on the
insertion torque of dental implants. Medicina Oral, Patología Oral y Cirugía Bucal.

[R27] Truong VM, Kim S, Kim J, Lee JW, Park YS (2022). Revisiting the Complications of Orthodontic Miniscrew. BioMed Res Int.

[R28] Nandini N, Kunusoth R, Alwala AM, Prakash R, Sampreethi S, Katkuri S (2022). Cylindrical Implant Versus Tapered Implant: A Comparative Study. Cureus.

[R29] Romero-Serrano M, Romero-Ruiz MM, Herrero-Climent M, Rios-Carrasco B, Gil-Mur J (2024). Correlation between Implant Surface Roughness and Implant
Stability: A Systematic Review. Dent J.

[R30] Quispe-López N, Martín-Martín S, Gómez-Polo C, Figueras-Alvarez O, Sánchez-Jorge MI, Montero J (2024). Primary and Secondary Stability Assessments of Dental Implants
According to Their Macro-Design, Length, Width, Location, and Bone Quality. Appl Sci.

[R31] Eini E, Yousefimanesh H, Ashtiani AH, Saki-Malehi A, Olapour A, Rahim F (2022). Comparing success of immediate versus delay loading of implants
in fresh sockets: a systematic review and meta-analysis. Oral Maxillofac Surg.

[R32] Maino BG, Di Blasio, Spadoni D, Ravanetti F, Galli C, Cacchioli A, et al (2017). The integration of orthodontic miniscrews under mechanical
loading: a pre-clinical study in rabbit. Eur J Orthod.

[R33] Jensen SW, Jensen ED, Sampson W, Dreyer C (2021). Torque Requirements and the Influence of Pilot Holes on
Orthodontic Miniscrew Microdamage. Appl Sci.

[R34] Azmi FNAM, Ying LS, Mohamed WAB, Hassan R (Available from:
http://proceedings.ui.ac.id/index.php/uiphm/article/view/4
).

[R35] McManus MM, Qian F, Grosland NM, Marshall SD, Southard TE (2011). Effect of miniscrew placement torque on resistance to miniscrew
movement under load. Am J Orthod Dentofacial Orthop.

[R36] Chen JC, Ko CL, Lin DJ, Wu HY, Hung CC, Chen WC (2019). In vivo studies of titanium implant surface treatment by
sandblasted, acid-etched and further anchored with ceramic of tetracalcium
phosphate on osseointegration. J Aust Ceram Soc.

[R37] Wagner J, Spille JH, Wiltfang J, Naujokat H (2022). Systematic review on diabetes mellitus and dental implants: an
update. Int J Implant Dent.

[R38] Sachelarie L, Scrobota I, Cioara F, Ghitea TC, Stefanescu CL, Todor L, et al (2025). The Influence of Osteoporosis and Diabetes on Dental Implant
Stability: A Pilot Study. Medicina (Mex).

[R39] Domingue D, Sinada N, White JR (2021). Digital surgical planning and placement of osseointegrated
implants to retain an auricular prosthesis using implant software with
cone-beam computed tomography and 3D-printed surgical guides: A case report. Clin Case Rep.

[R40] Putra RH, Cooray U, Nurrachman AS, Yoda N, Judge R, Putri DK, et al (2023). Radiographic alveolar bone assessment in correlation with primary
implant stability.

[R41] Zumstein T, Sennerby L (2016). A 1-Year Clinical and Radiographic Study on Hydrophilic Dental
Implants Placed with and without Bone Augmentation Procedures. Clin Implant Dent Relat Res.

[R42] Kulczyk T, Czajka-Jakubowska A, Przystańska A (2018). A Comparison between the Implant Stability Quotient and the
Fractal Dimension of Alveolar Bone at the Implant Site. BioMed Res Int.

[R43] Singh N, Rajesh N, Ramesh A (2024). Assessment of implant stability with resonance frequency analysis
and changes in the thickness of keratinized tissue and crestal bone level
using cone-beam computed tomography in two-stage implants: A
three-dimensional clinicoradiographic study. J Indian Soc Periodontol.

[R44] Antony DP, Thomas T, Nivedhitha M (2020 Apr 19 [cited 2025 May 7]). Two-dimensional Periapical, Panoramic Radiography Versus
Three-dimensional Cone-beam Computed Tomography in the Detection of
Periapical Lesion After Endodontic Treatment.

[R45] Ozarslanturk S, Ozturk HP, Senel B, Avsever H, Ozen T (2018 Feb 27 [cited 2025 May 7]). What Surprises Lie Beneath a Panoramic Radiograph in Dental Implant
Planning. Dent Adv Res [Internet].

[R46] Chopra A, Singh R, Thukral R, Mittal A (2021). Cone-beam computed tomography in implant dentistry: Radiation
dose, field of view, and use guidelines. Imaging Sci Dent.

[R47] Song D, Shujaat S, de Faria, Huang Y, Politis C, Lambrichts I, et al (2021). Diagnostic accuracy of CBCT versus intraoral imaging for
assessment of peri-implant bone defects. BMC Med Imaging.

[R48] Arisan V, Karabuda ZC, Avsever H, Özdemir T (2013). Conventional Multi-Slice Computed Tomography (CT) and Cone-Beam
CT (CBCT) for Computer-Assisted Implant Placement Part I: Relationship of
Radiographic Gray Density and Implant Stability. Clin Implant Dent Relat Res.

[R49] Bornstein MM, Horner K, Jacobs R (2017). Use of cone beam computed tomography in implant dentistry:
current concepts, indications and limitations for clinical practice and
research. Periodontol 2000.

[R50] Vasoglou G, Stefanidaki I, Apostolopoulos K, Fotakidou E, Vasoglou M (2022). Accuracy of Mini-Implant Placement Using a Computer-Aided
Designed Surgical Guide, with Information of Intraoral Scan and the Use of a
Cone-Beam CT. Dent J.

[R51] Miotk N, Schwindling FS, Zidan M, Juerchott A, Rammelsberg P, Hosseini Z, et al (2023). Reliability and accuracy of intraoral radiography, cone beam CT,
and dental MRI for evaluation of peri-implant bone lesions at zirconia
implants − an ex vivo feasibility study. J Dent.

[R52] Van Dessel, Nicolielo LFP, Huang Y, Slagmolen P, Politis C, Lambrichts I, et al (2016). Quantification of bone quality using different cone beam computed
tomography devices: Accuracy assessment for edentulous human mandibles. Eur J Oral Implantol.

[R53] Neldam CA, Lauridsen T, Rack A, Lefolii TT, Jørgensen NR, Feidenhans’l R, et al (2015). Application of high resolution synchrotron micro-CT radiation in
dental implant osseointegration. J Cranio-Maxillofac Surg.

[R54] Kaaber L, Matzen LH, Schropp L, Spin-Neto R (2024). Low-dose CBCT protocols in implant dentistry: a systematic
review. Oral Surg Oral Med Oral Pathol Oral Radiol.

[R55] Dos Santos, Jacobs R, Quirynen M, Huang Y, Naert I, Duyck J (2011). Peri-implant bone tissue assessment by comparing the outcome of
intra-oral radiograph and cone beam computed tomography analyses to the
histological standard: Peri-implant bone tissue assessment. Clin Oral Implants Res.

[R56] Commission E, Energy DG (2012).

[R57] Centeno ACT, Fensterseifer CK, Chami VDO, Ferreira ES, Marquezan M, Ferrazzo VA (2022). Correlation between cortical bone thickness at mini-implant
insertion sites and age of patient. Dent Press J Orthod.

[R58] Uday N, Prashanth KP, Kumar A (2017). CBCT evaluation of interdental cortical bone thickness at common
orthodontic miniscrew implant placement sites. Int J Appl Dent Sci.

[R59] Kalra S, Tripathi T, Rai P, Kanase A (2014). Evaluation of orthodontic mini-implant placement: a CBCT study. Prog Orthod.

[R60] Becker K, Unland J, Wilmes B, Tarraf NE, Drescher D (2019). Is there an ideal insertion angle and position for orthodontic
mini-implants in the anterior palate A CBCT study in humans. Am J Orthod Dentofacial Orthop.

[R61] Hung KF, Ai QYH, Wong LM, Yeung AWK, Li DTS, Leung YY (2023). Current Applications of Deep Learning and Radiomics on CT and
CBCT for Maxillofacial Diseases. Diagnostics.

[R62] Mugri MH (2025). Accuracy of Artificial Intelligence Models in Detecting
Peri-Implant Bone Loss: A Systematic Review. Diagnostics.

[R63] Rezallah NNF, Luke AM (2025). Evaluating Micro-computed Tomography in Dental Implant
Osseointegration: A Systematic Review and Meta-analysis. Acad Radiol.

[R64] Goswami T (2012).

[R65] Setiawan K, Primarti RS, Sitam S, Suridwan W, Usri K, Latief FDE (2024). Microstructural Evaluation of Dental Implant Success Using
Micro-CT: A Comprehensive Review. Appl Sci.

[R66] Galletti F, D’Angelo T, Fiorillo L, Lo Giudice, Irrera N, Rizzo G, et al (2024). Micro-CT Structure Analysis on Dental Implants: Preliminary In
Vitro Trial. Prosthesis.

[R67] Sotova C, Yanushevich O, Kriheli N, Grigoriev S, Evdokimov V, Kramar O, et al (2023). Dental Implants: Modern Materials and Methods of Their Surface
Modification. Materials.

[R68] Putri A, Pramanik F, Azhari A (2023). Micro Computed Tomography and Immunohistochemistry Analysis of
Dental Implant Osseointegration in Animal Experimental Model: A Scoping
Review. Eur J Dent.

[R69] Schreck J, Niehoff JH, Saeed S, Kroeger JR, Lennartz S, Laukamp KR, et al (2025). Dental implant artifacts: Evaluation of photon counting
CT-derived virtual monoenergetic images in combination with iterative metal
artifact reduction algorithms. Eur J Radiol.

[R70] Vanden Broeke, Grillon M, Yeung AWK, Wu W, Tanaka R, Vardhanabhuti V (2021). Feasibility of photon-counting spectral CT in dental
applications—a comparative qualitative analysis. BDJ Open.

[R71] Li B, Hu Y, Xu S, Li B, Inscoe CR, Tyndall DA, et al (2024). Low-cost dual-energy CBCT by spectral filtration of a dual focal
spot X-ray source. Sci Rep.

[R72] Zanon C, Pepe A, Cademartiri F, Bini C, Maffei E, Quaia E, et al (2024). Potential Benefits of Photon-Counting CT in Dental Imaging: A
Narrative Review. J Clin Med.

[R73] Wajer R, Wajer A, Kazimierczak N, Wilamowska J, Serafin Z (2024). The Impact of AI on Metal Artifacts in CBCT Oral Cavity Imaging. Diagnostics.

[R74] Troiano G, Rapani A, Fanelli F, Berton F, Caroprese M, Lombardi T, et al (2024). Inter and intra-operator reliability of Lekholm and Zarb
classification and proposal of a novel radiomic data-driven clustering for
qualitative assessment of edentulous alveolar ridges. Clin Oral Implants Res.

[R75] Calabrò E, Lisnic T, Cè M, Macrì L, Rabaiotti FL, Cellina M (2024). Dynamic Digital Radiography (DDR) in the Diagnosis of a Diaphragm
Dysfunction. Diagnostics.

[R76] Thirunavukkarasu R, Mani B, Nirupama C, Muralidharan D, Tamizhmani J, Prasanth C (2022). CBCT in orthodontics: A boon for the millennial generation. Int J Health Sci.

[R77] Filonenko VV (2023). Determination of density of bone structures of the maxillo-facial
region in clinical practice. Exp Clin Med.

